# Differential effects of antiretrovirals on microbial translocation and gut microbiota composition of HIV-infected patients

**DOI:** 10.7448/IAS.20.1.21526

**Published:** 2017-03-09

**Authors:** María J. Villanueva-Millán, Patricia Pérez-Matute, Emma Recio-Fernández, José M. Lezana Rosales, José A. Oteo

**Affiliations:** ^a^ HIV and Associated Metabolic Alterations Unit, Infectious Diseases Department, Center for Biomedical Research of La Rioja (CIBIR), Logroño, La Rioja, Spain; ^b^ Infectious Diseases Department, Hospital San Pedro, Logroño, La Rioja, Spain

**Keywords:** HIV infection, antiretroviral therapy, gut microbiota, bacterial diversity, microbial translocation, inflammation

## Abstract

**Introduction:** Increased bacterial translocation and alterations to gut microbiota composition have been described in HIV infection and contribute to immune activation and inflammation. These effects persist despite combined antiretroviral therapy (cART). However, the contribution of different cART combinations has not yet been investigated. The aim of this study was to analyse the long-term effects of different combinations of cART on bacterial translocation and gut microbiota composition in HIV-infected patients.

**Methods:** We carried out a cross-sectional study of 45 HIV-infected patients on cART, classified as nucleoside reverse transcriptase inhibitors (NRTIs)+ protease inhibitors (PIs) (n = 15), NRTIs+ non-nucleoside reverse transcriptase inhibitors (NNRTIs) (n = 22), and NRTIs+ integrase strand transfer inhibitors (INSTIs) (n = 8). Untreated HIV-infected patients (n = 5) and non-infected volunteers (n = 21) were also included. Soluble markers of bacterial translocation and inflammation were measured and gut microbiota composition was analysed using 16S rDNA pyrosequencing (Illumina MiSeq).

**Results:** The NRTIs+INSTIs regimen was associated with levels of systemic inflammation that were similar to uninfected controls. The reduction in faecal bacterial diversity induced by HIV infection was also restored by this regimen. HIV infection was more closely related to changes in lower taxonomic units and diversity rather than at the phylum level. The NRTIs+PIs regimen showed the highest reduction in bacterial species, whereas NRTIs+INSTIs induced a minor loss of bacterial species and a significant increase in others.

**Conclusions:** Our study demonstrated that INSTI-based ART was associated with levels of systemic inflammation and microbial diversity similar to that of uninfected controls. The role of INSTIs other than raltegravir needs to be further investigated. Patients on the NRTIs+PIs regimen presented the highest reduction in bacterial species compared with other antiretrovirals and naive patients. Thus, different cART regimens are associated with diverse profiles in gut microbiota composition. Longitudinal and functional studies are needed to better understand these findings.

## Introduction

Depletion of gastrointestinal CD4^+^ T cells during HIV infection is followed by alterations to lymphoid tissue architecture, integrity and loss of function of the mucosal barrier [1]. Loss of immune protection of the intestinal mucosa allows translocation of microbial products (such as lipopolysaccharides) into the lamina propria of the gastrointestinal tract and, eventually, into the systemic circulation [2–4]. This is known as bacterial translocation (BT), which was firstly described in HIV-infected patients in 2006 [5,6]. These bacterial products induce a significant increase in proinflammatory cytokine production via toll-like receptors, contributing to immune activation and inflammation [2,4]. The degree of BT is linked to the severity of HIV progression independent of viremia and several studies have demonstrated that increased BT and proinflammatory cytokines are partially responsible for HIV-related comorbidities [7–11].

The gastrointestinal microbiota (GM) is the richest and most diverse system of bacteria and other microorganisms that live symbiotically with the host [12]. Dysbiosis (an imbalance in microbiota composition) has been linked with chronic inflammation associated with several pathologies such as obesity, diabetes, inflammatory bowel disease (IBD), and cardiovascular disease. Alterations in GM in HIV infection are also present and have been related to the development of non-AIDS events [13,14]. The resilience mechanisms are not able to restore GM composition and persist despite combined antiretroviral therapy (cART) [15–17]. A very recent study addressed the role of different families of ART (non-nucleoside reverse transcriptase inhibitors (NNRTIs) vs. protease inhibitors (PIs)) on GM composition, although no differences were observed [17]. To our knowledge, other cART combinations have not yet been investigated. Thus, the aim of this study was to analyse the long-term effects of different combinations of cART on BT and GM composition in HIV-infected patients with undetectable viral load. We also tested whether these families of antiretrovirals were able to restore GM composition compared with an uninfected population.

## Methods

### Study design

This was a cross-sectional study carried out in HIV-infected people from San Pedro’s Hospital, Logrono, Spain. Patients were selected for the study according to the following criteria: on cART (HIV+(cART)) for at least one year and with viral load <20 cop/mL for at least 6 months (n = 45). Thus, all subjects included were immune responders. These patients were classified depending on family treatment: nucleoside reverse transcriptase inhibitors and PIs (NRTIs+PIs) (n = 15), NRTIs and NNRTIs (NRTIs+NNRTIs) (n = 22), and NRTIs with integrase strand transfer inhibitors (NRTIs+INSTIs) (n = 8). We also included untreated HIV-infected patients (HIV+(naive)) (n = 5) (average viral load of 54,010 cop/mL (3,550–71,800 cop/mL)) and non-infected volunteers (controls) (n = 21) as our reference group. The control population was matched for age, gender and body mass index with the HIV-infected group as these factors are known to influence GM composition [6,14]. For both HIV patients and controls, the following exclusion criteria applied: <18 years old; pregnant women; patients treated with antibiotics, anti-inflammatory drugs, corticosteroids, immunosuppressive drugs, or probiotics in the last 3 months; individuals with kidney, coeliac, or inflammatory disease, thyroid disorders, neoplasms, history of intestinal surgery (except appendectomy or cholecystectomy), IBD (even if inactive), chronic pancreatitis, or any syndrome related to an intestinal malabsorption. Patients receiving statins were also excluded [18].

In case of coinfection, degree of liver fibrosis was evaluated non-invasively using the FibroScan® (Echosens, Paris, France) method. Patients were classified according to the METAVIR scoring system (F0, no fibrosis; F1, portal fibrosis without septa; F2, portal fibrosis and few septa; F3, numerous septa without cirrhosis; F4, cirrhosis) [19].

The study was performed following the Helsinki Declaration and was approved by the Committee for Ethics in Clinical Research in La Rioja (CEICLAR) (23 April 2013, reference number 121). All participants provided their written informed consent.

## Soluble markers of BT, inflammation, and endothelial damage

Plasma and serum samples were collected after a 12 h fast. Samples were centrifuged and stored at −80ºC for subsequent analyses. Serum levels of soluble CD14 (sCD14) and lipopolysaccharide-binding protein (LBP) were assessed using an enzyme-linked immunosorbent assay (ELISA) (R&D, Minneapolis, USA and Hycult Biotech, Uden, The Netherlands, respectively). Intercellular adhesion molecule (ICAM) and vascular cell adhesion molecule (VCAM) plasma levels were quantified by a Human Premixed Multi-Analyte Kit (Luminex, Minneapolis, USA) and serum levels of interleukin 6 (IL-6) were determined by ELISA (R&D, Minneapolis, USA).

## DNA extraction from stool samples and 16S rDNA sequencing

Both fresh stool and plasma samples were collected at enrolment. Faecal DNA was extracted using the DNeasy Blood & Tissue Kit (Qiagen, Venlo, Netherlands) and purity and concentration were subsequently determined by a Nanodrop spectrophotometer 1000 (Thermo Scientific, USA).

Samples were amplified for the 16S rDNA hypervariable sequence V4 using primers (515F-806R) [20]. Sequencing was performed using the Illumina MiSeq Instrument (two readings per 150 base pairs) (Illumina, INC, San Diego, CA, USA). Computational analysis was carried out by Era7 Bioinformatics (Granada, Spain). Briefly, the first step was to assemble the two reads obtained from the Illumina technology. The computational tool FLASh was used to extend the reads prior to assembly to obtain a larger sequence for a more specific taxonomic assignment of the reads which was carried out based on direct similarity of each read, one-by-one, compared with any sequence 16S included in the Ribosomal Database Project [21] through the BLAST (Basic Local Alignment Search Tool) programme. Two different taxonomic assignment approaches were used: BBH (Best Blast Hit: each read was assigned to the taxon corresponding to the BBH over a threshold of similarity) and LCA (Lowest Common Ancestor: adopted by advanced tools of metagenomic analysis such as the last version of MEGAN 24). The median number of sequences assigned per patient was 89,030.21 with the LCA method, whereas 100,888.61 sequences were obtained with the BBH method. Both α- and β-diversity were analysed: α-diversity is a measure of sample-level species richness, with healthy subjects typically exhibiting more species richness than those with intestinal conditions [16], whereas β-diversity describes inter-subject similarity of microbial composition and facilitates identification of broad differences between samples [22].

## Statistical analysis

Results are presented as mean ± standard error of the mean (SEM). P values <0.05 were considered statistically significant. Categorical variables were analysed using the Chi-square test or Fisher’s exact test. Normal distribution of quantitative variables was checked using the Shapiro-Wilk test. Comparisons between three or more groups were analysed by one way ANOVA followed by a Bonferroni *post hoc* test or by Kruskal-Wallis test followed by Dunns post-tests. Comparisons between two groups were performed with Unpaired *t* test or U Mann-Whitney. Relationships between variables were analysed by calculating Pearson’s rank correlation coefficients. Statistical analysis was performed using SPSS 19.0 (SPSS® Inc. Chicago, IL, USA) and GraphPad Prism 6 (GraphPad Prism®, La Jolla, California, USA). α-diversity was calculated and presented as four indices (number of observed species, Margalef’s diversity index, Chao 1 and Alpha index) using R (version 3.2.2; The R foundation for Statistical Computing, Vienna, Austria), whereas β-diversity was assessed using the web server METAGENassist [23]. Data obtained from β-diversity were statistically analysed using the Wilcoxon rank-sum non-parametric test. A principal component analysis (PCA) was also developed. Results are plotted according to the first two principle components [23].

## Results

### Clinical and demographic characteristics of participants

Table 1 shows the main characteristics of the population. All participants were Caucasian. Sixty per cent of the HIV+(naive) patients presented over 500 nadir CD4^+^ cells/mm^3^, whereas only 11.1% of the patients using cART showed nadir CD4^+^ cells above 500 cells/mm^3^ (p = 0.024). There were no differences concerning the different families of cART used although patients in the NRTIs+INSTIs group had a higher incidence of AIDS (p = 0.013). More than 50% of the patients using cART presented coinfection with hepatitis C virus (p = 0.054), with no differences between the treatments. HIV patients were infected for an average of 16 years. Total time using cART (including the therapy evaluated in this study) averaged 13 years. No differences were observed for the total length on treatment, although a slightly longer time on treatment was observed in the INSTI group, showing significant differences compared with the NRTIs+NNRTIs group (p = 0.048).

**Table 1.** Characteristics of healthy, uninfected controls and HIV-infected patients.

		HIV+			HIV+(cART)			
	Control	HIV+(naive)	HIV+(cART)		NRTIs+PIs	NRTIs+NNRTIs	NRTIs+INSTIs	
No. of patients	21	5	45	Overall p value^1^	15	22	8	Overall p value^2^
Gender (male)	11/21 (52.38%)	4/5 (80%)	30/45 (66.7%)	0.449	11/15 (73.33%)	13/22 (59.09%)	6/8 (75%)	0.639
Age (years)	48.81 ± 2.65	44.2 ± 4.87	48.68 ± 0.98	0.541	51.27 ± 1.47	47.29 ± 1.62	47.5 ± 1.43	0.166
Body mass index (kg/m^2^)	27.41 ± 1.2	25.78 ± 2.67	24.33 ± 0.89	0.140	23.09 ± 1.2	25.7 ± 1.65	23.9 ± 1.74	0.697
CD4 nadir count (cells/mm^3^)	-	<200: 0/5 (0%)	<200: 19/45 (42.22%)	0.142	<200: 9/15 (60%)	<200: 6/22 (27.27%)	<200: 4/8 (50%)	0.135
	-	200–500: 2/5 (40%)	200–500: 21/45 (46.67%)	1	200–500: 6/15 (40%)	200–500: 12/22 (54.55%)	200–500: 3/8 (37.5%)	0.673
	-	>500: 3/5 (60%)	>500: 5/45 (11.11%)	0.024	>500: 0/15 (0%)	>500: 4/22 (18.18%)	>500: 1/8 (12.5%)	0.180
CD4 count (cells/mm^3^)	-	200–500: 2/5 (40%)	200–500: 12/45 (26.7%)	0.611	200–500: 6/15 (40%)	200–500: 4/22 (18.18%)	200–500: 2/8 (25%)	0.369
	-	>500: 3/5 (60%)	>500: 33/45 (73.3%)	0.611	>500: 9/15 (60%)	>500: 18/22 (81.82%)	>500: 6/8 (75%)	0.361
T4/T8 index	-	0.62 ± 0.09	0.92 ± 0.05	0.068	0.83 ± 0.09	0.94 ± 0.08	1 ± 0.14	0.482
Time since diagnosis of HIV infection (years)	-	3.20 ± 0.73	16.51 ± 1.14	<0.001	18.53 ± 2.11	13.95 ± 1.55	19.75 ± 1.95	0.111
AIDS	-	0/5 (0%)	20/45 (44.4%)	0.075	9/15 (60%)	5/22 (22.73%)^a^	6/8 (75%) ^b^	0.013
Coinfection with hepatitis B virus	-	0/5 (0%)	1/45 (2.2%)	1	1/15 (6.67%)	0/22 (0%)	0/8 (0%)	0.513
Coinfection with hepatitis C virus	-	0/5 (0%)	23/45 (51.11%)	0.054	10/15 (66.67%)	8/22 (36.36%)	5/8 (62.5%)	0.140
Mode of transmission	-	IVDU: 1/5 (20%)	IVDU: 17/45 (37.78%)	0.642	IVDU: 7/15 (46.67%)	IVDU: 5/22 (22.73%)	IVDU: 5/8 (62.5%)	0.099
	-	HS: 2/5 (40%)	HS: 17/45 (37.78%)	1	HS: 6/15 (40%)	HS: 10/22 (45.45%)	HS: 1/8 (12.5%)	0.285
	-	MSM: 1/5 (20%)	MSM: 2/45 (4.44%)	0.276	MSM: 0/15 (0%)	MSM: 2/22 (9.09%)	MSM: 0/8 (0%)	0.669
	-	IVDU/HS: 0/5 (0%)	IVDU/HS: 1/45 (2.22%)	1	IVDU/HS: 0/15 (0%)	IVDU/HS: 0/22 (0%)	IVDU/HS: 1/8 (12.5%)	0.179
	-	Unknown: 1/5 (20%)	Unknown: 8/45 (17.78%)	1	Unknown: 2/15 (13.33%)	Unknown: 5/22 (22.73%)	Unknown: 1/8 (12.5%)	0.878
Degree of hepatic fibrosis	-	F0–F1: 5/5 (100%)	F0–F1: 29/45 (64.44%)	0.163	F0–F1: 6/15 (40%)	F0–F1: 17/22 (77.27%)	F0–F1: 6/8 (75%)	0.078
	-	F2: 0/5 (0%)	F2: 5/45 (11.11%)	1	F2: 4/15 (26.67%)	F2: 1/22 (4.55%)	F2: 0/8 (0%)	0.096
	-	F3: 0/5 (0%)	F3: 8/45 (17.78%)	0.577	F3: 3/15 (20%)	F3: 4/22 (18.18%)	F3: 1/8 (12.5%)	1
	-	F4: 0/5 (0%)	F4: 3/45 (6.67%)	1	F4: 2/15 (13.33%)	F4: 0/22 (0%)	F4: 1/8 (12.5%)	0.172
Advanced degree of hepatic fibrosis	-	F2–F4: 0/5 (0%)	F2–F4:16/45 (35.56%)	0.163	F2–F4: 9/15 (60%)	F2–F4: 5/22 (22.73%)	F2–F4: 2/8 (25%)	0.079
Time on cART (years)	-	-	13.55 ± 1	-	14.47 ± 1.73	11.29 ± 1.42	17.75 ± 1.71^b^	0.054
Time on the last cART (years)	-	-	5.31 ± 0.41	-	5.73 ± 0.84	5.32 ± 0.59	4.50 ± 0.68	0.497

F0, no fibrosis; F1, portal fibrosis without septa; F2, portal fibrosis and few septa; F3, numerous septa without cirrhosis; F4, cirrhosis) [19]; HIV+(cART), HIV-infected patients on combined antiretroviral therapy for at least one year or more and with viral load <20 copies/mL for at least six months; HIV+(naive), untreated HIV-infected patients; HS, heterosexual; IVDU, intravenous drug user; IVDU/HS, intravenous drug user and multiple heterosexual contacts; MSM, men who have sex with men; NRTIs+INSTIs, nucleoside reverse transcriptase inhibitors and integrase strand transfer inhibitors; NRTIs+NNRTIs, nucleoside reverse transcriptase inhibitors and non-nucleoside reverse transcriptase inhibitors; NRTIs+PIs, nucleoside reverse transcriptase inhibitors and protease inhibitors. Quantitative data are presented as mean ± SEM, whereas qualitative data are indicated as percentage. Overall p value^1^ was obtained comparing controls vs. HIV+(naive) and HIV+(cART). Overall p value^2^ was obtained by comparing the three treatments. ^a^p < 0.05 vs. NRTIs+PIs, ^b^p < 0.05 vs. NRTIs+NNRTIs.

## Bacterial translocation, inflammation and endothelial markers

**Figure 1.**
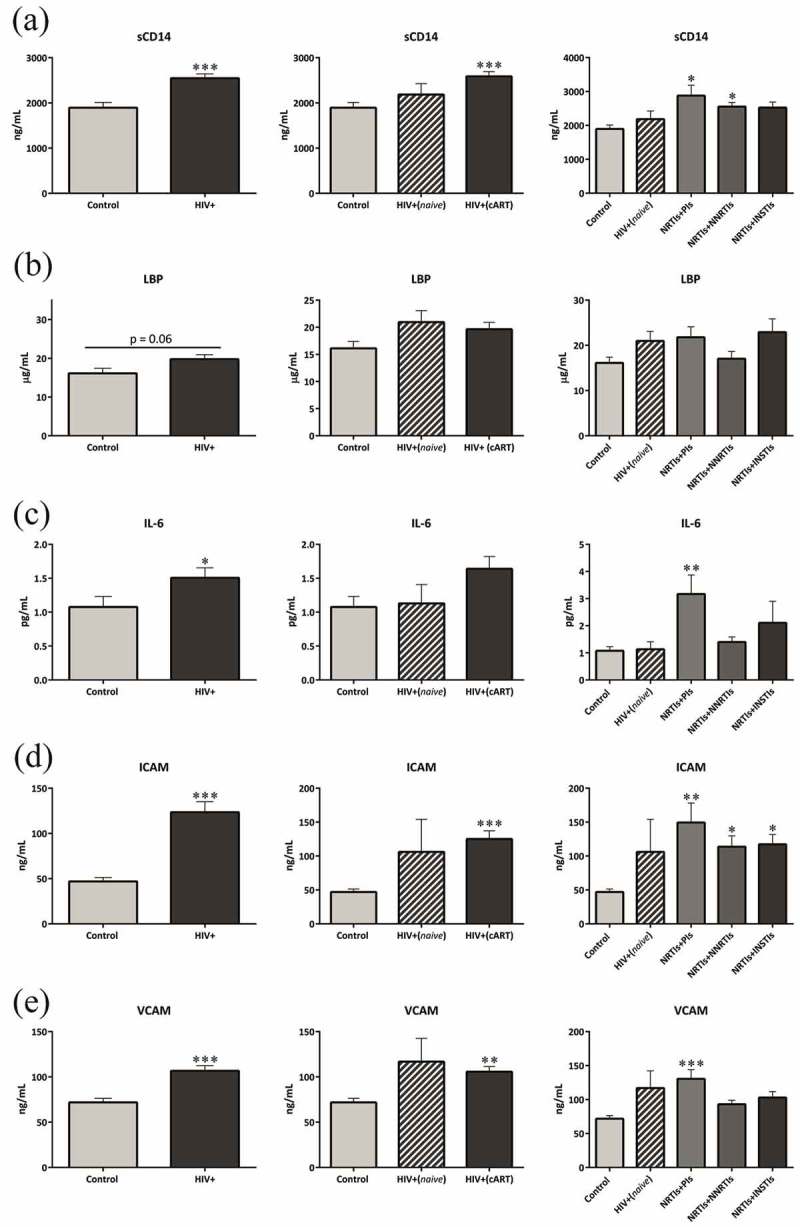
Increased bacterial translocation, inflammation and endothelial damage. LBP, lipopolysaccharide-binding protein; HIV+, includes both HIV-infected patients on antiretroviral treatment and those untreated; HIV+(cART), HIV-infected patients on combined antiretroviral therapy for at least one year or more and with viral load <20 copies/mL for at least six months; HIV+(naive), untreated HIV-infected patients; ICAM, intercellular adhesion molecule; IL-6, interleukin 6; sCD14, soluble CD14; VCAM, vascular cell adhesion molecule. Each bar represents the mean ± SEM. p < 0.05 was considered significant. *p < 0.05; **p < 0.01; ***p < 0.001 vs. control (uninfected patients).

sCD14 plasma levels were significantly increased in HIV+ patients compared with controls (p = 0.0003), especially in those patients using cART (NRTIs+PIs, p = 0.034 and NRTIs+NNRTIs, p = 0.011 vs. controls, respectively). However, patients using NRTIs+INSTIs presented similar sCD14 plasma levels to the controls (Figure 1a), although no significant differences were observed between the cART regimens. No changes were observed in LBP plasma levels (Figure 1b). IL-6, ICAM and VCAM plasma levels were significantly increased in HIV+ patients (p < 0.05, p < 0.001 and p < 0.001, respectively), especially in those on NRTIs+PIs treatment compared with the controls (p = 0.005, p = 0.005 and p < 0.001, respectively). ICAM values were also significantly increased in patients using NRTIs+NNRTIs (p = 0.011 vs. controls) and NRTIs+INSTIs (p = 0.020 vs. controls), although the increase was less potent than that observed with the NRTIs+PIs regimen (Figure 1c–e). A positive association was found between sCD14 and IL-6 and VCAM in all HIV-infected patients (p = 0.006 and p = 0.023, respectively, data not shown).

A significant increase was observed in LBP plasma levels in coinfected patients compared with non-coinfected participants (p = 0.0007) and also compared with controls (p = 0.005). No significant differences were observed between both HIV-infected groups when sCD14 was quantified (Supplementary Figure 1).

## Gut microbiota diversity and composition

**Figure 2.**
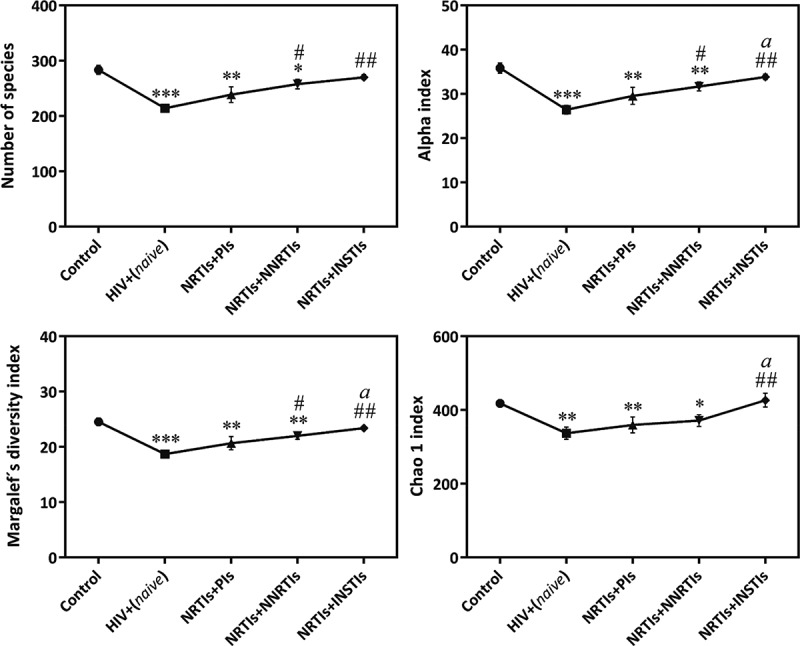
α-diversity measurements in control group compared with untreated HIV infected patients and using different cART. HIV+(naive), untreated HIV-infected patients; NRTIs+INSTIs, nucleoside reverse transcriptase inhibitors and integrase strand transfer inhibitors; NRTIs+NNRTIs, nucleoside reverse transcriptase inhibitors and non-nucleoside reverse transcriptase inhibitors; NRTIs+PIs, nucleoside reverse transcriptase inhibitors and protease inhibitors combination. Four indices were used to represent the α-diversity: number of species, Alpha, Margalef’s diversity and Chao 1. Each bar represents the mean ± SEM. p < 0.05 was considered significant. *p < 0.05; **p < 0.01; ***p < 0.001 vs. control, ^#^p < 0.05; ^##^p < 0.01 vs. HIV+(naive), ^a^p < 0.05 vs. NRTIs+PIs.

HIV infection dramatically decreased α-diversity (p = 0.0006–p = 0.003) (Figure 2). Patients in the NRTIs+INSTIs group showed a similar α-diversity profile to the controls and a significant increase compared with the HIV+(naive) group (p = 0.006–p = 0.008). The combination of NRTIs+NNRTIs was also able to partially restore the α-diversity (p = 0.026–p = 0.033 vs. naive).

Coinfected patients showed a lower α-diversity than the controls. No significant differences were observed among coinfected and non-coinfected patients (data not shown). Coinfected patients with advanced hepatic fibrosis (F2–F4) showed a trend towards reduced α-diversity compared with patients with mild hepatic fibrosis (F0–F1) (p = 0.098–p = 0.078, data not shown).

Concerning GM composition (β-diversity), the most abundant phyla in gut were Bacteroidetes and Firmicutes. Thus, approximately 73% of the bacteria detected in gut belong to these phyla (74.11%, 73.14% and 74.2% for control, HIV+(naive) and HIV+(cART), respectively) (Table 2). No significant differences were observed in the most abundant phyla in the gut when HIV patients were compared with controls. Only an increase in the abundance of Proteobacteria and, consequently, the ratio of Proteobacteria/Firmicutes, was observed in HIV-infected patients on cART (p = 0.021; p = 0.023 vs. control, respectively). A significant decrease (p = 0.049) in the abundance of Firmicutes was observed in HIV-infected patients using NRTIs+PIs compared with controls. Lentisphaerae, Euryarchaeota, Synergistetes, and Fusobacteria represented around 0.18% of the bacteria. The differences observed in the abundance of these phyla are shown in Table 2.

**Table 2.** Relative abundance of major phyla present in the gut in control and HIV-infected patients.

		HIV positive			HIV-positive (cART)			
	Control	HIV-positive (*naive*)	HIV-positive (cART)	Overall *p* value^1^	NRTIs + PIs	NRTIs + NNRTIs	NRTIs + INSTIs	Overall *p*-value^2^
Firmicutes	42.86 ± 1.56	36.04 ± 5.88	39.17 ± 1.47	0.281	36.72 ± 2.78	40.54 ± 2.02	40.02 ± 3.40	0.505
Bacteroidetes	31.25 ± 2.46	37.10 ± 2.81	35.03 ± 1.68	0.347	37.68 ± 2.71	34.80 ± 2.49	30.69 ± 4.02	0.513
Proteobacteria	2.96 ± 0.35	4.69 ± 1.56	5.15 ± 0.54 *	0.068	4.05 ± 0.65	5.92 ± 1.04	5.95 ± 1.11	0.386
* α-Proteobacteria	0.43 ± 0.12	0.02 ± 0.01 *	0.29 ± 0.07	0.086	0.34 ± 0.09	0.18 ± 0.08	1.63 ± 0.76 ^b^	0.028
* β-Proteobacteria	0.79 ± 0.2	1.33 ± 0.47	1.28 ± 0.2	0.478	1.56 ± 0.41	1.10 ± 0.24	1.28 ± 0.49	0.837
* γ-Proteobacteria	0.10 ± 0.02	0.06 ± 0.04	0.12 ± 0.03	0.179	0.06 ± 0.02	0.34 ± 0.14	0.17 ± 0.09	0.470
* δ-Proteobacteria	0.49 ± 0.07	0.51 ± 0.14	0.91 ± 0.10 *	0.023	0.70 ± 0.14	0.96 ± 0.17	1.15 ± 0.14 ^a^	0.176
Actinobacteria	0.96 ± 0.17	1.50 ± 0.09	1.44 ± 0.22	0.229	3.44 ± 0.94	1.24 ± 0.21	1.11 ± 0.23	0.419
Lentisphaerae	0.05 ± 0.02	0.02 ± 0.02	0.08 ± 0.04 *	0.037	0.01 ± 0.007	0.11 ± 0.08	0.11 ± 0.08	0.167
Euryarchaeota	0.08 ± 0.05	0.0004 ± 0.0002	0.02 ± 0.01 **	0.006	0.01 ± 0.009	0.03 ± 0.02	0.04 ± 0.04	0.426
Synergistetes	0.03 ± 0.01	0.0004 ± 0.0004	0.04 ± 0.03 **	0.023	0.10 ± 0.09	0.005 ± 0.004	0.02 ± 0.02	0.615
Fusobacteria	0.002 ± 0.0008	0.002 ± 0.001	0.04 ± 0.03	0.850	0.007 ± 0.006	0.02 ± 0.01	0.15 ± 0.15	0.266
Ratio Bacteroidetes/Firmicutes	0.76 ± 0.09	1.21 ± 0.29	0.97 ± 0.07	0.124	1.06 ± 0.11	0.83 ± 0.09	0.85 ± 0.16	0.268
Ratio Proteobacteria/Firmicutes	0.08 ± 0.01	0.14 ± 0.04	0.13 ± 0.01 *	0.068	0.09 ± 0.01	0.12 ± 0.02	0.16 ± 0.04 ^a^	0.245
Ratio Actinobacteria/Firmicutes	0.02 ± 0.004	0.04 ± 0.01	0.05 ± 0.007	0.094	0.10 ± 0.02	0.04 ± 0.007	0.03 ± 0.004	0.388

HIV+(cART), HIV-infected patients on combined antiretroviral therapy for at least one year or more and with viral load <20 copies/mL for at least six months; HIV+(naive), untreated HIV-infected patients; NRTIs+INSTIs, nucleoside reverse transcriptase inhibitors and integrase strand transfer inhibitors; NRTIs+NNRTIs, nucleoside reverse transcriptase inhibitors and non-nucleoside reverse transcriptase inhibitors; NRTIs+PIs, nucleoside reverse transcriptase inhibitors and protease inhibitors. Data are presented as mean ± SEM. Overall p value^1^ was obtained by comparing controls vs. HIV+(naive) and HIV+(cART). Overall p value^2^ was obtained by comparing the three treatments. *p < 0.05; **p < 0.01 vs. control (non-infected subjects). ^a^p < 0.05 vs. NRTIs + PIs, ^b^p < 0.05 vs. NRTIs + NNRTIs.

At the class level, a significant increase was observed in the relative abundance of δ-Proteobacteria in HIV+(cART) (p = 0.025 vs. controls). This increase was more evident in the NRTIs+INSTIs group (p = 0.013 vs. control). A decrease in the abundance of α-Proteobacteria was observed in naive patients (p = 0.028 vs. control) and cART was able to restore the abundance of this bacterial class (Table 2).

Coinfection also induced some slightly changes in GM at phyla, class, genera and species levels compared with non-coinfected patients (Supplementary Table 1 and 2).

One of the goals of this study was to test whether different families of ART currently used in clinical practice were able to induce a complete restoration of GM composition after HIV infection. Thus, we compared GM composition (at lower taxonomic units) of HIV+(cART) patients against the uninfected subjects (controls). A combination of NRTIs+INSTIs produced a pronounced increase in bacteria belonging to Desulfovibrionales and Selenomonadales orders, Desulfovibrionaceae and Lachnospiraceae families and *Desulfovibrio* genus, whereas only a significant depletion in the abundance of unclassified Clostridiales order was observed. Patients using NRTIs+NNRTIs showed an increased abundance of Coriobacteriales order, Coriobacteriaceae and Lachno-spiraceae bacterial families, as well as *Pseudomononas* genus, whereas a lower abundance of Bacteroidales order, Bacteroidaceae family and *Streptococcus* genus was observed. Finally, patients using NRTIs+PIs showed a significant increase in the presence of Clostridiales order, Lachnospiraceae family and *Eggerthella* genus, and a significant reduction in Actinomycetales, Pseudom-onadales, and Sphingomonadales orders, Eubacteriaceae and Prevotellaceae families, and *Prevotella, Pseudom-onas* and *Solobacterium* genera (Table 3). More specifically, NRTIs+INSTIs and NRTIs+PIs patients showed a higher abundance of *Blautia* sp. 3. In addition, a significant increase in the abundance of *Flavonifractor plautii* was observed in NRTIs+PIs patients, whereas an increase in the levels of *Parabacteroides merdae* was observed in NRTIs+INSTIs patients. A higher number of bacterial species were reduced in HIV patients using NRTIs+PIs (10 species), whereas patients using NRTIs+NNRTIs or NRTIs+INSTIs regimens showed a lower abundance of only six bacterial species compared with controls (Table 3).

**Table 3.** Combined antiretroviral therapy modified the relative abundance of taxonomical groups (order, family, genus, species) compared with uninfected-controls subjects.

NRTIs+INSTIs, nucleoside reverse transcriptase inhibitors and integrase strand transfer inhibitors; NRTIs+NNRTIs, nucleoside reverse transcriptase inhibitors and non-nucleoside reverse transcriptase inhibitors; NRTIs+PIs, nucleoside reverse transcriptase inhibitors and protease inhibitors. Red represents a significant increase in the relative abundance of the taxonomical groups compared with the control group, whereas blue represents a significant decrease.

**Figure 3.**
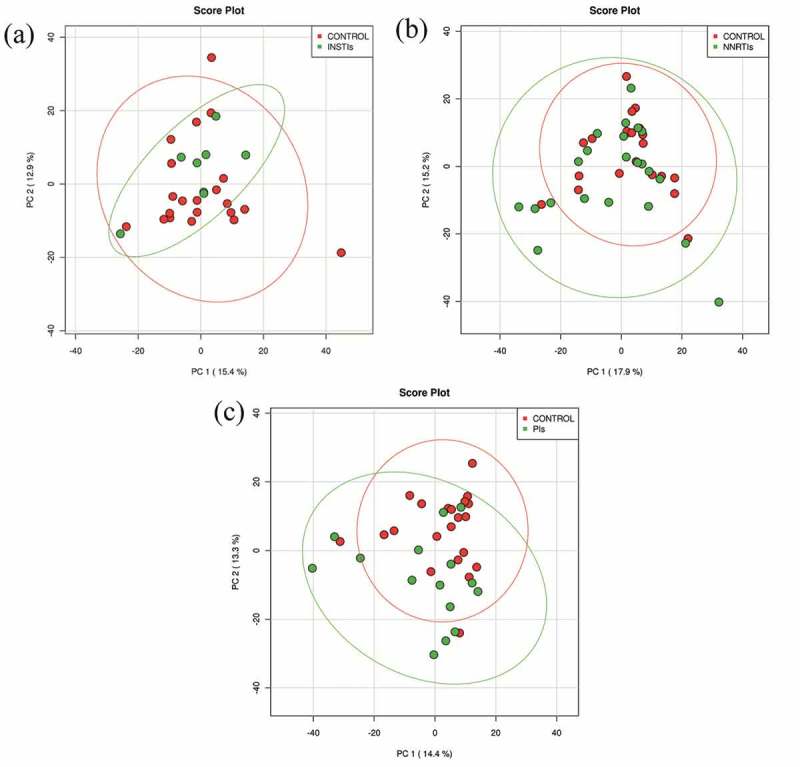
Principal component analysis of different combined antiretroviral therapy compared with the control/uninfected individuals. INSTIs, HIV-infected patients using nucleoside reverse transcriptase inhibitors and integrase strand transfer inhibitors; NNRTIs, HIV-infected patients using nucleoside reverse transcriptase inhibitors and non-nucleoside reverse transcriptase inhibitors; PIs, HIV-infected patients using nucleoside reverse transcriptase inhibitors and protease inhibitors. Results are plotted according to the first two principle components. Each circle represents a sample: red circles represent the uninfected volunteers, while green circles represent the HIV-infected patients using a combined antiretroviral therapy. The clustering of samples is represented by their respective 95% confidence interval ellipse. (a) PCA of the HIV-infected patients using NRTIs+INSTIs combination vs. non-infected subjects (accounting for 28.3% of the total variation (Component 1 = 15.4% and Component 2 = 12.9%)). (b) PCA of the HIV-infected patients using NRTIs+NNRTIs combination vs. non-infected subjects (accounting for 33.1% of the total variation (Component 1 = 17.9% and Component 2 = 15.2%)). (c) PCA of the HIV-infected patients using NRTIs+PIs combination vs. non-infected subjects (accounting for 27.7% of the total variation (Component 1 = 14.4% and Component 2 = 13.3%)).

Figure 3 shows a PCA where the NRTIs+INSTIs cluster is represented inside the control cluster, in contrast with the diagrams obtained for the other combinations of cART compared with the controls.

## Discussion

Our study demonstrates for the first time that INSTI-based ART is associated with levels of systemic inflammation, sCD14 plasma levels, and microbial diversity similar to uninfected controls, suggesting a healthier gut and potentially fewer HIV-related complications.

It is important to note that the patient cohort comprised long-term evolution-treated HIV-infected patients with an effective ART therapy for at least one year. Our study demonstrated that sCD14 levels (a marker of BT) were significantly increased in HIV-infected patients, still evident despite cART, as previously described [15,24–27]. Surprisingly, levels of sCD14 in NRTIs+INSTIs patients did not differ from those observed in the control/uninfected group. Furthermore, NRTIs+INSTIs patients also showed similar levels of IL-6, VCAM and ICAM to the controls, suggesting that this regimen is able to counteract the increased BT induced by HIV infection and also diminish systemic inflammation, potentially reducing future HIV-related complications (such as cardiovascular events) triggered by BT and inflammation. In this context, several studies have previously demonstrated that starting treatment with INSTIs and/or the intensification or switching to this regimen is associated with a favourable effect on HIV-related immune activation and also with inhibition of CD4-T cell depletion [28–32] which is in line with our findings. It is also important to note than although an increase in BT and inflammation was observed in HIV-infected patients compared with the controls, such a difference was only significant in HIV-patients using cART. This could be due to the low number of naive patients recruited in this study which makes difficult to reach statistical significance. A potential impact of cART on inflammation markers cannot be ruled out as Hileman et al. demonstrated that patients who were switched to cART showed different effects on immune activation, which may affect vascular inflammation markers [30].

It is obvious that various factors such as diet, geographical distribution, stress, etc. could also contribute to the gut alterations that occur during HIV infection. Among them, coinfection with hepatitis C virus (HCV) and/or hepatitis B virus could have significant clinical relevance. In our cohort, around 50% of HIV patients were also coinfected with these hepatotrophic viruses. Although no significant differences were found in the percentage of HIV-coinfected patients under the three different treatments, we decided to analyse the effect of such coinfection independently on cART on BT as well as on GM composition. Our study demonstrates that coinfected patients presented higher levels of BT than non-coinfected. However, these differences were only observed in LBP levels and not sCD14, which has been suggested to be a more relevant biomarker of disease progression as it reflects the host response to products of BT [33]. Thus, these discrepancies could be explained by the different markers used. Measurement of more than one marker is recommended in order to have a broader view of what is really happening in gut [33,34].

Although HIV infection has been associated with a reduced bacterial diversity [16,17,35], others have observed a significant increase in HIV+(naive) patients compared with HIV+(cART) (Supplementary Table 3) [36]. Our results show a significant and clear collapse in α-diversity in HIV+(naive) compared with the controls as occurs in other pathologies such as obesity [6]. Compared with controls, we observed a more pronounced decrease in untreated patients than in those using cART, suggesting that cART is able to partially restore the bacterial diversity in gut. A study by Lozupone et al. also observed that long-term cART is able to partially restore α-diversity to the values obtained in HIV-negative individuals; however, in contrast with our findings, they observed a significant increase in α-diversity in chronic HIV+(naive) patients compared with controls [36]. These discrepancies could be due to the different indices used to compare α-diversity. Concerning the effects of different antiretrovirals, our study demonstrates that NRTIs+INSTIs patients present a microbial diversity similar to the controls, which highlights the ability of this regimen to counteract the actions of HIV infection on gut bacterial richness. The superior capacity of cART with INSTIs to restore GM diversity may be due to the fact that INSTIs induce a greater reduction in proviral DNA, which could lead to rapid immunologic reconstitution [37–39].

Regarding GM composition, HIV infection and usage of different ART did not translate to significant changes at higher taxonomic levels, suggesting that HIV infection could be more closely related to changes in lower taxonomic units (specific bacteria) and diversity rather than at the phylum level, as occurs in other metabolic pathologies [6]. These findings contrast with other studies that observed changes in some of the most abundant phyla when HIV-infected patients (both naive and using cART) were compared with healthy individuals [16,40]. Thus, McHardy et al. showed a significant decrease in Firmicutes phylum in HIV+(naive) patients and intermediate depletion in HIV+(cART) compared with controls, whereas we only observed a slightly depletion in this phylum in both HIV+(naive) patients and patients using NRTIs+PIs compared with the controls [16]. In contrast, we observed a significant increase in the relative abundance of Proteobacteria in HIV+(cART) with no differences among the different ART used, whereas others have not reported any differences [41]. A potential explanation for these discrepancies could be the type of sample used (rectal mucosal biopsies vs. faeces). There is no agreement regarding the best sample to use for these determinations, and, therefore, this is an issue that needs further investigation. However, it is worth mentioning that the majority of the changes observed at lower taxonomic levels in our study were detected in taxonomic groups belonging to Firmicutes phylum and, especially, to the Clostridiales class. Thus, seven bacterial species were found to be depleted in NRTIs+PIs patients, four were depleted in NRTIs+NNRTIs patients, and five depleted in NRTIs+INSTIs patients. In contrast, within the Clostridiales class, Lachnospiraceae family, one of the major taxonomic groups of the human GM known to degrade complex polysaccharides to short-chain fatty acids to be used as energy by the host, was significantly increased with cART, suggesting that these patients are more efficient from an energetic point of view [42]. *Eubacterium eligens* and *Ruminococcus flavefaciens* were two species whose abundance was significantly depleted by all treatments used. Nowak et al. also observed a decrease in *Eubacterium* genus in HIV-infected patients compared with healthy subjects, especially after introducing cART [17]. Of interest, a decrease was observed in *Faecalibacterium prausnitzii* in NRTIs+PIs patients. *F. prausnitzii* is a beneficial intestinal commensal bacterium with known anti-inflammatory properties; thus, the decrease observed could imply loss of protection and persistent inflammation [43,44]. These findings are in agreement with the increased BT observed in these patients. In addition, a reduction in the abundance of several bacteria normally present in gut (*Roseburia inulinivorans* and *Roseburia intestinal*) has also been observed in the patients on this regimen, corroborating the loss of richness observed compared with the controls. Finally, patients using NRTIs+INSTIs showed a lesser reduction in bacteria from the Clostridiales class. In addition, a reduction in *Desulfovibrio* sp. 6 was observed. This bacterium belongs to the *Desulfovibrio* genus, known to produce hydrogen sulphide, a compound that can be toxic to human cells. Surprisingly, this genus was found to be increased in our patients. Similarly, higher frequencies of this genus has been found in other pathologies such as IBD and also in HIV infection [36,45,46]. In contrast, Nowak et al. found a significant increase in viraemic patients compared with controls, although the relative abundance of this genus decreased after introduction of ART [17]. These discrepancies could be due to the fact that Nowak et al. did not include patients on the NRTIs+INSTIs regimen [17].

A higher number of patients on INSTIs presented with AIDS compared with the other groups. The usage of INSTIs in these patients was considered a very effective rescue therapy. In fact, similar CD4 counts were observed when compared with the other groups. Therefore, differences in GM composition could not be due to the CD4 counts. In fact, these patients presented a GM composition similar to uninfected volunteers, which highlights the interest of such findings and merits further investigation.

This study has several limitations. Important aspects that could have an impact in GM composition have not been controlled in this study, such as the exact composition of the diet, stress conditions and HIV acquisition (heterosexual, men who have sex with men, intravenous drug user, etc.) [47–50]. Moreover, it would be interesting to compare the GM composition in the HIV+(naive) patients with different CD4 count ranges; however, this comparison could not be performed because of the small sample size and because patients with a CD4 count <400 cells/mm^3^ were not included in this study. The effects of coinfection are of interest and could influence GM composition [51]. However, no significant differences were found in the percentage of HIV coinfected patients on the three different treatments. Thus, we could suggest that the potential effects of HCV in GM are similar in the three groups under cART and, therefore, the changes observed in our study are mainly due to the drugs and not to HCV. However, we also performed a metagenomic analysis, separating HIV-infected patients depending on coinfection and independently of cART. As previously mentioned with BT, very slight effects were observed on BT and major phyla abundance. Some changes at genus and species levels were observed. Thus, our results demonstrated a different microbiota composition in HIV coinfected patients when compared with non-coinfected patients, at least at lower taxonomic levels, and for this reason, the impact of such coinfection could not be discarded and should be taken into account in these types of studies. Another limitation is that all patients using NRTIs+INSTIs were only treated with raltegravir, as it was the first INSTI approved for clinical practice and the only one available when patients were recruited for this study. Thus, it is unknown whether elvitegravir or dolutegravir would have similar effects and deserves further investigation. Finally, our study also included a very limited number of untreated HIV-infected patients. However, the comparison among controls and HIV+(naive) has been previously described and our purpose was to analyse the effects of different cART. In fact, our study includes more patients on cART than others published to date [15–17,35,36,41]. Our study provides a clear description of GM composition in HIV-infected patients compared with a healthy population and specifically investigates in-depth the impact of different antiretrovirals in order to better understand which regimen is able to restore GM composition and, therefore, resist the actions of HIV infection on BT and subsequent immune activation, disease progression and future complications.

## Conclusions

HIV infection is closely associated with changes in lower taxonomic units and diversity rather than at the phylum level. The NRTIs+PIs regimen showed the highest reduction in bacterial species, which could suggest a significant loss of diversity and increased dysbiosis. In contrast, NRTIs+INSTIs increased the abundance of several bacterial orders, families, genera, and bacterial species and induced a minor loss of bacterial species suggesting a healthier gut, which, in turn, could contribute to the lower inflammation and BT observed. However, further studies are needed to analyse whether INSTIs other than raltegravir have similar effects. In addition, longitudinal and functional studies are needed to ensure that the changes observed in this study are clinically significant.

## Notes

To access the supplementary material to this article please see Supplementary Files under Article Tools online.
